# Insight into antioxidant and anti-inflammatory effects of marine bacterial natural exopolysaccharide (EPSSM) using carrageenan-induced paw edema in rats

**DOI:** 10.1038/s41598-024-53502-5

**Published:** 2024-03-01

**Authors:** Mohamed E. El awady, Sahar S. Mohamed, Mostafa M. Abo Elsoud, Manal G. Mahmoud, Mai M. Anwar, Mahgoub M. Ahmed, Ashraf Eltaher, Sameh Magdeldin, Ashraf Attallah, Ali E. Elhagry, Sayeda A. Abdelhamid

**Affiliations:** 1 Microbial Biotechnology Department, National Research Centre, El-Buhouth St. 33 Dokki, Cairo, Egypt; 2 Department of Biochemistry, National Organization for Drug Control and Research (NODCAR)/Egyptian Drug Authority (EDA), Cairo, Egypt; 3 Molecular Drug Evaluation Department, National Organization for Drug Control and Research (NODCAR), Cairo, 12553 Egypt; 4 Senior research associate at 57357 hospital Master of life science informatics at Bonn university, Bachelor of pharmaceutical sciences, Cairo University, Cairo, Egypt; 5 Proteomics and Metabolomics Research Program, Basic Research Unit, Research Department, Children’s Cancer Hospital Egypt 57357, Cairo, 11441 Egypt; 6 Department of Physiology, Faculty of Veterinary Medicine, Suez Canal University, Ismailia, 41522 Egypt; 7 Microbial Genetics Department, National Research Center, El-Buhouth St. 33 Dokki, Cairo, Egypt; 8 Microbiology Department, Faculty of Science, Al-Azhar University, Cairo, Egypt

**Keywords:** Biochemistry, Biotechnology, Microbiology

## Abstract

Inflammation is a part of the body’s intricate biological reaction to noxious stimuli and defensive reactions. So, the aim of this investigation was to study the anti-inflammatory activity of exopolysaccharide (EPSSM) using carrageenan-induced paw edema in rats. A halophilic bacterial strain was isolated from marine sediments in the Red Sea in Egypt. The isolate has been visually and physiologically recognized, as well as by analyzing its 16S rRNA gene, which confirms *Kocuria* sp. clone Asker4. This particular isolate can be referenced using the accession number OL798051.1. EPSSM was subjected to purification and fractionation by a DEAE-cellulose column. Preliminary chemical analysis of EPSSM indicated that the monosaccharides were fructose, glucuronic acid, and xylose, with 2.0, 0.5, and 1.0, respectively. The antioxidant potential of EPSSM was investigated, and it was discovered that the level of activity increased independently of the concentrations, reaching a maximum threshold of 94.13% at 100 µg/mL of EPSSM for 120 min. Also, EPSSM at 50 mg/kg orally produced a significant anti-inflammatory effect on the carrageenan model at 2, 3, and 4 intervals. The EPSSM intervention resulted in reductions in the levels of catalase and superoxide dismutase enzymes, as well as a decrease in glutathione. Furthermore, the levels of nitric oxide, lipid peroxidation, and reactive oxygen species resulting from carrageenan-induced edema showed a significant reduction subsequent to the administration of EPSSM. Moreover, the findings indicated that the protein expression levels of cyclooxygenase-2 and interleukin-6 were reduced following treatment with EPSSM, resulting in a reduction of paw edema.

Within the realm of natural ecosystems, an abundant array of pharmacological agents can be found, with the origins of numerous contemporary medications being rooted in natural products. Natural products encompass chemical substances that exist in either standardized extracts or as pure compounds. These compounds are derived from living creatures and have biological impacts on other organisms. The vast chemical variety found in natural products presents significant potential for the development of novel pharmaceuticals. Natural materials are present in multicellular organisms, including plants, animals, and marine sources such as sponges and snails, as well as in unicellular organisms like bacteria and yeasts. These materials possess medicinal qualities^1,2^. Bacteria that produce EPS are commonly found in marine habitats and can be obtained from several sources, such as the water column, sediments, and animals. Bacteria exhibiting the ability to synthesize polymers with unique shapes and exceptional properties have been discovered in unconventional habitats, such as severe settings^3,4^. The natural compounds derived from microorganisms, including omega-3 fatty acids, cyclic peptides, antimicrobial peptides, oligosaccharides, and polysaccharides, have demonstrated the potential to mitigate inflammation. These compounds can be readily integrated into the diet without causing any detrimental consequences^2^. Exopolysaccharides (EPSs), which are an essential component of bacterial biofilm, are highly valuable commercial biopolymers that serve as a protective coating for the bacteria. It has been found that the EPSs generated by the halophiles exhibit favorable biotechnological features^5,6^. In the last several years, there has been significant interest within the disciplines of biochemistry and pharmacology in the bioactive polysaccharides derived from natural sources. The health-promoting properties of exopolysaccharides, including their anticancer, antiulcer, immunomodulating, antioxidant, and cholesterol-lowering effects, have made them valuable components for use in the food business^7,8^. Probiotics derived from unicellular organisms such as bacteria and yeasts are known to inhabit the gastrointestinal tract (GIT) and have potential applications in the management of inflammatory bowel disease and the treatment of infectious diarrhea^9^. Various commensal microorganisms, including *Lactobacilli, Bifidobacteria, Bacteroides fragilis, Bacillus* sp., and others, have the ability to synthesize natural chemicals that possess immunomodulatory and anti-inflammatory characteristics. Anti-inflammatory cytokines encompass a diverse range of immune regulatory molecules that exhibit a reaction to proinflammatory cytokines, including IL-1β, IL-6, IL-8, INF-γ, and TNF-α. The group of anti-inflammatory cytokines encompasses several molecules, such as the antagonist IL-1 receptor, IL-4, IL-6, IL-10, IL-11, and IL-13^10^. During the process of inflammation, the production of cyclooxygenase-2 (COX-2) and nitric oxide (NO) radicals occurs. Nitric oxide (NO) is synthesized by the enzyme known as oxygen nitric oxide synthase (iNOS) through the reaction between oxygen and L-arginine. The activity of this enzyme is subject to regulation during the inflammatory process. Similar to inducible nitric oxide synthase (iNOS), cyclooxygenase-2 (COX-2) is tightly regulated in its response to infectious pathogens, atherosclerosis, and various cancers. The control of inducible nitric oxide synthase (iNOS) and cyclooxygenase-2 (COX-2) gene expression during inflammation is regulated by the pro-inflammatory transcription factor, nuclear factor kappa B (NF-κB)^11^. Certain members of the toll-like receptor (TLR) family, particularly TLR4, have been identified as the primary receptors for lipopolysaccharide (LPS). This work involved the isolation of a marine bacteria strain that exhibits a high synthesis of EPS from the Red Sea in Egypt. The bacterium was subsequently identified as *Kocuria* sp. The process of isolating, fractionating, and characterizing the EPS was conducted. Moreover, the present study investigates the effects of EPS on anti-inflammatory responses and oxidative stress in rats.

## Materials and methods

### Sampling and culture medium

Samples of sea sediment were obtained from the beach of Porto El Sokhna, located in the Red Sea region of Egypt. The sediment samples were obtained at depths ranging from 2 to 3 m. Each sediment sample was individually put into a medium with the following composition per liter: glucose (20 g), CaCO_3_ (0.1 g), NH_4_NO_3_ (0.8 g), K_2_HPO_4_ (0.6 g), KH_2_PO_4_ (0.05 g), MnSO_4_·4H_2_O (0.1 g), yeast extract (1.0 g), and agar (15 g). This medium was utilized for the purposes of isolating, purifying, and temporarily storing the collected sediments. The medium was produced by combining on-site collected natural seawater with distilled water in a volumetric ratio of 3:1. All media were incubated at 37 °C. By observing for slimy mucous colonies on the plate for three days, mucous colony formation was investigated^12^.

### Isolation of a bacterium that produces exopolysaccharides (EPS)

The samples underwent processing with the subsequent method: A wet sediment sample weighing 1 g was diluted with 100 ml of sterile sea water. Following the mixing process, after which it was subjected to serial dilution. A volume of 1 mL from each dilution was evenly distributed onto the agar plate medium. The Petri dishes were placed in an incubator set at a temperature of 37 °C and periodically examined for the presence of bacterial growth. Then, the colonies present on the plates were subjected to purification using the streak plate method^13^.

### Screening of EPS producing isolate

A total of thirty colonizing strains were observed to possess glossy and viscous surfaces on the growth medium. This observation signifies the synthesis of EPS by bacterial organisms^14^. The strains were cultured in 25 mL shake flasks using a liquid medium composed of 750 mL of seawater and 250 mL of distilled water per liter. The media consisted of (g/L) sucrose (20), yeast extract (2), peptone (4). The cultures were subjected to incubation in a shaker for a duration of three days at a temperature of 37 °C and 120 rpm. The culture medium underwent centrifugation at 5000 rpm for 30 min to eliminate cells. Subsequently, the supernatant subjected to deproteinization using TCA 5%^15^. The EPS was obtained by adding 5 volumes of 95 or 98% ethanol and afterward collecting it using centrifugation at 5000 rpm for 20 min at 4 °C. Then, the precipitate washed with acetone and dried under vacuum. The pellet obtained was subsequently dissolved in a minimal amount of deionized water and subjected to three rounds of dialysis utilizing dialysis tubing with a molecular weight cutoff (MWCO) of 3500 Da. The dialysis process was carried out by immersing the tubing in running tap water for duration of 24 h. The dialyzed solution was subjected to a fractional precipitation method, wherein quantities of totally cold ethanol (1, 2, 3, and 4) were employed for treatment. The main portion was obtained through the use 2 volume of ethanol and subjected to vacuum drying at a temperature of 40 °C, utilizing a single volume of ethanol with a purity of 95 or 98%.

### Identification of the promising isolate

The present study aims to undertake a comprehensive analysis of the morphological and physiological characteristics. The bacterial isolate with the highest potency, referred to as SM, was identified by a series of morphological, physiological, and biochemical investigations in accordance with the guidelines outlined in Bergey’s Manual of Determinative Bacteriology^16^.

### Genetic identification of the potent isolate by 16srRNA

The genomic DNA was extracted from the strain (SM) using the method published by Li et al.^17^ Subsequently, PCR amplification and sequencing of the 16S rRNA gene were conducted using the protocol outlined in the same study. The process of isolating intact genomic DNA was carried out via the NucleoSpin^®^ Tissue Kit, manufactured by (Macherey–Nagel). The amplification of the 16S rDNA gene sequence was conducted using a universal primer sourced from Integrated DNA Technology in India. The forward primer sequence utilized in this study was 5′-AGAGTTTGATCATGGCTCAG-3′, while the reverse primer sequence employed was 5′-GGTTACCTTGTTACGACTT-3′. The alignment and comparison of the 16S rRNA sequences were conducted by utilizing the NCBI Basic Local Alignment Search Tool (BLAST-n) program available at http://www.ncbi.nlm.nih.gov/BLAST. The resulting sequences were subsequently submitted to GenBank in order to get accession numbers. The alignment of 16S rRNA sequences was conducted using the Bio-edit tool, with the exclusion of areas containing nucleotides that were deemed ambiguous. The phylogenetic trees were generated using the neighbour-joining statistical method in MEGA X software (https://www.megasoftware.net/dload_win_gui).

### Bioinformatics

Both F and R Sequences are aligned on NCBI blast (https://blast.ncbi.nlm.nih.gov/Blast.cgi) where a set of aligned genes is obtained with the *blastn* option and a set of aligned proteins is obtained with the *blastx* option for each. The set of aligned gene and protein sequences then separately underwent multiple alignments using *the CLUSTAL W option* of the GenomeNet database (https://www.genome.jp/tools-bin/clustalw) and a phylogenetic tree is created for each and viewed with the help of the ETE toolkit tree viewer (http://etetoolkit.org/treeview/?treeid=0f765eb27910baa1678948b68ee60b39&algid=f2194283a82229238a5a98a555afcd59). Aligned proteins from *blastx* were simultaneously ordered by total score, and the highest scoring protein was selected to generate its 3D structure via the *SWISS-MODEL*** (**https://swissmodel.expasy.org/**)** and *I-TASSER* (https://zhanggroup.org/I-TASSER/) web servers, respectively. *SVA06781* showed the highest total score in both F and R sequence protein alignments. The *SWISS-MODEL* generates multiple models with different templates for the selected protein, which are ranked by coverage (https://swissmodel.expasy.org/interactive/JWvYqE/models). The top-ranked model in terms of coverage further goes for structure assessment with Ramachandran’s plot (https://swissmodel.expasy.org/assess/JWvYqE/01).

The *I-TASSER* models for the selected protein were ranked by C-score (confidence) and the top 5 are displayed. The *COFACTOR* method, available on the I-TASSER server (https://zhanggroup.org/COFACTOR/), is utilized to predict the most significant protein–ligand binding sites, enzyme commission numbers, active sites, and gene ontology terms. This is achieved through the application of structure comparison and analysis of protein–protein networks. For further details, please refer to the output of the I-TASSER server at http://zhanglab.ccmb.med.umich.edu/I-TASSER/output/S745549/.

*COACH* meta-server approach of the I-TASSER server (https://zhanggroup.org/COACH/), then combines multiple function annotation results (on ligand-binding sites) from the COFACTOR, TM-SITE, and S-SITE programs (https://zhanggroup.org/COACH/output/CH2099413779/).

### Optimization and statistical modeling of EPSSM production

The Box-Behnken-Behnkenhas been built based on five variables, namely: sucrose, peptone, yeast extract, pH, and incubation temperature. The testing range for each variable has been shown in Table 1. The design resulted in 46 runs. EPSSM production (g/l) was considered as the response of the design and analyzed with regard to the term (*P* value < 0.05). Variables with a *P* value < 0.05 were considered significant model terms. Design-Expert software (Stat-Ease Inc., Minneapolis, MN, USA, version 7.0.0) was used for building, evaluation, and data analysis. Design-Expert software was used for maximization of EPSSM production and prediction of the optimum levels of the variables with the highest desirability.

**Table 1:** Summary of the design variables used for Box–Behnken design and their levels.

Factor	Name	Units	Study range	
			Low	High
A	Sucrose	g/l	10	50
B	Peptone	g/l	2	6
C	Yeast extract	g/l	1	5
D	pH	Unit	5	8
E	Incubation temperature	°C	30	40

### Ion exchange chromatography

Diethylaminoethyl-cellulose (DEAE-cellulose) was prepared. In this procedure, the fraction displaying the highest level of activity from the preceding step was concentrated and adsorbed onto a DEAE-cellulose column (70 × 1.5 cm) that had been pre-equilibrated with distilled water. The EPSSM that was not absorbed was subjected to a washing process using distilled water, while the EPSSM that was absorbed was separated using a stepwise elution method with NaCl concentrations ranging from 0.2 to 1.0 M. A volume of 1.0 mL per minute was collected, and a small aliquot was subjected to analysis using the phenol–sulfuric acid method^18^. The fractions exhibiting activity were combined, subjected to dialysis using deionized water, and subsequently precipitated with ethanol following concentration.

### Chemical analysis of EPSSM

The quantification of total sugars was conducted using the phenol_H_2_SO_4_ method as described by DuBois et al.^18^, with glucose serving as the standard. The measurement of uronic acid was conducted using the m_hydroxybiphenyl technique, as described by Filisetti-Cozzi and Carpita^19^, glucuronic acid was employed as the standard for this analysis. The determination of sulfate was conducted subsequent to hydrolysis, whereby formic acid with a concentration of 85% was employed at a temperature of 100 °C for a duration of 5 h. The turbidimetric method, as described by Dodgson and Price^20^ was utilized for this purpose, with sodium sulfate serving as the standard. The monosaccharide composition of EPSSM was analyzed using high-performance liquid chromatography (HPLC) with a Shimadzu Shim-Pack SCR-101N column (7.9 mm × 30 cm). Deionized water was employed as the mobile phase, and the flow rate was set at 0.5 mL/min °C, as described by Sudhamani et al.^21^. The process of identifying sugar involved comparing it with genuine samples of sugar. The measurement of the weight-average molecular weight (*Mw*) and number-average molecular weight (*Mn*) of EPS was conducted using an Agilent 1100 High Performance Liquid Chromatography (HPLC) system equipped with a Refractive Index (*RI*) Detector. The EPS was dissolved in a 2 mL amount of solvent and later underwent filtration using a 0.45 μm filter before injection. The determination of the polydispersity index (*PI*) involves the calculation of the ratio between the weight-average molecular weight (*Mw*) and the number-average molecular weight (*Mn*)^22,23^. The Fourier Transform Infrared (FT-IR) spectra of EPSSM was analyzed using a Bucker Scientific 500-IR FTIR spectrophotometer manufactured by Bucker Co. in Ettlingen, Germany. The spectrum was recorded within the wavenumber range of 4000–400 cm^−1^. The EPSSM that had been purified was subjected to grinding with KBr powder of spectroscopic grade, followed by compression into pellets for the purpose of conducting FTIR measurements^24^.

### The evaluation of antioxidant activity

#### DPPH scavenging activity

The antioxidant activity of EPSSM (20, 40, 60, 80, and 100 µg/mL) was measured using the DPPH assay for 30, 60, 90, and 120 min. Brand-Williams et al.^25^, and the scavenging activity was computed as follows:

$${\text{Scavenging}}\;{\text{ability}}\left( \% \right) = \left( {{{\text{A}}_{517\;{\text{of}}\;{\text{control}}}} - {{\text{A}}_{517\;{\text{of}}\;{\text{sample}}}}/{{\text{A}}_{517\;{\text{of}}\;{\text{control}}}}} \right) \times 100.$$

### Reducing power activity

The method described by Oyaizu^26^, A mixture consisting of 2.5 mL of a 0.2 M phosphate buffer solution with a pH of 6.6 and 2.5 mL of a K_3_Fe(CN)_6_ solution with a concentration of 1% w/v is combined with 1.0 mL of EPSSM solution, which has concentrations of 100–500 µg/mL, dissolved in distilled water. The resultant mixture is subjected to incubation at a temperature of 50 °C for a duration of 20 min. Subsequently, 2.5 mL of trichloroacetic acid (10%) are added. The mixture was subjected to centrifugation at a speed of 3000 rpm for 10 min in order to separate and collect the upper layer of the solution, which amounted to 2.5 mL. This collected upper layer was then combined with 2.5 mL of distilled water and 0.5 mL of FeCl_3_ solution, which had a concentration of 0.1%. The measurement of absorbance is thereafter conducted at a wavelength of 700 nm using a blank sample as a reference.

### Anti-inflammatory activity of EPSSM

#### Animals

All the conducted animal experimental studies were approved by the Animal Ethical Committee of the National Organization of Drug Control and Research (NODCAR) (Certificate Number: NODCAR/II/51/2022) in accordance with the relevant guidelines and regulations in addition to ARRIVE guidelines.

### Experimental design

#### Experimental animals

The current study employed male albino rats (160 ± 20 g) sourced from the laboratory stock colony of the National Organization for Drug Control and Research (NODCAR). The animals were housed under typical environmental conditions for one week before the start of the experiment, with unrestricted access to water and a standardized diet. They were individually housed in stainless-steel enclosures, maintained at a consistent ambient temperature ranging from 20 to 22 °C, and with a relative humidity of approximately 55%.

A group of 36 male albino rats was divided into six subgroups, each consisting of six rats. The initial step involved using a German-manufactured computerized digital caliper to measure the thickness of the hind paw of each animal. EPSSM samples were prepared by dissolving them in distilled water. The rats were orally administered EPSSM at a dosage of 50 mg/kg. One hour after these treatments, each rat received an administration of a 1% carrageenan suspension (0.1 mL per animal) in the dorsal region of its left hind paw, following the protocol by Winter et al.^27^. The thickness of the hind paw of each rat was measured at five specific time intervals: 1, 2, 3, 4, and 5 h after the carrageenan administration.

#### Biochemical investigation

All employed rats were euthanized by decapitation under the influence of 3% halothane anesthesia in 40% Oxygen-balance Nitrogen. A two-gram sample of paw tissues was obtained and subjected to a rinsing process using ice-cold distilled water. Subsequently, the tissues were promptly immersed in a solution consisting of three times their volume of cold 1.15% KCl, supplemented with 0.2% Triton X-100. The resulting mixture was homogenized, and the homogenate underwent centrifugation at a force of 8000 g for 10 min to obtain the supernatant. The supernatant was then stored at a temperature of − 20 °C^28^.

### Determination of oxidative stress parameters

#### Detection of malondialdehyde (MDA)

The assessment of MDA content was conducted using the thiobarbituric acid (TBA) test, as outlined by Ohkawa et al.^29^. The reaction between MDA and TBA leads to the formation of a complex with a distinct color. To estimate the concentration of malondialdehyde (MDA), the absorbance at a wavelength of 532 nm was measured. The specific activity is quantified as nm/g tissue.

#### The quantification of reactive oxygen species (ROS) levels

The estimation of ROS was carried out following the methodology described by Vrablic et al.^30^. In this study, we employed a modified iteration of a previously established assay to assess the intracellular conversion of nitro-blue tetrazolium (NBT) into formazan by superoxide anion.

#### Measurement of Nitric Oxide (NO)

The estimation of NO was conducted by Wang et al.^31^ utilizing the Griess reaction. In summary, Griess reagent was introduced to a portion of the supernatants, resulting in the formation of a colored product, which was subsequently measured at a wavelength of 540 nm. The quantification of NO was performed using a standard curve.

#### Determination of reduced glutathione (GSH)

The levels of glutathione (GSH) were determined using the methodology described by Beutler et al.^32^. In brief, the homogenate was subjected to deproteinization using a 10% trichloroacetic acid solution, followed by centrifugation at a speed of 3500 rpm for 10 min. The supernatant, measuring 50 μL, was combined with a solution containing disodium hydrogen phosphate at a concentration of 0.32 mol/L and 5,5′-dithiobis 2-nitrobenzoic acid (DTNB) at a concentration of 0.04%. The spectrophotometric measurement was conducted at a wavelength of 412 nm to quantify the yellow-hued compound resulting from the reaction between GSH and DTNB. The outcomes were reported in terms of glutathione (GSH) concentration measured in mmol/g of tissue.

#### Evaluation of superoxide dismutase (SOD) activity

The determination of superoxide dismutase (SOD) activity in kidney homogenate was conducted in accordance with the methodology outlined by Masayasu and Hiroshi^33^. The approach employed in this study involves the generation of superoxide anions through the autoxidation of pyrogallol. The resulting superoxide anion is then detected using nitroblue tetrazolium (NBT) to form formazan, which can be quantitatively measured. The level of formazan color development serves as an indicator of the amount of superoxide anion scavenged by superoxide dismutase (SOD). The activity of superoxide dismutase (SOD) is quantified and reported as U/g tissue.

#### Measurement of catalase (CAT)

The measurement of catalase (CAT) activity was conducted in accordance with the methodology outlined by Osumi and Hashimoto^34^. This involved assessing the hydrolysis of hydrogen peroxide (H_2_O_2_) and quantifying the subsequent reduction in absorbance at a wavelength of 240 nm. The measurements were performed over duration of 3 min at a temperature of 25 °C. The activity of CAT is quantified in terms of U/g of tissue.

#### Protein expression of COX-2 and IL-6

The levels of COX-2 and IL-6 were assessed by employing commercially available ELISA kits (BioSource International, Inc., Camarillo, CA, USA) as per the manufacturer's instructions. The analysis was conducted on the supernatant.

### Statistical analysis

The statistical analyses were conducted using the SPSS software, employing a one-way ANOVA followed by the post-hoc Duncan test. A significance level of *P* < 0.05 was deemed to indicate statistical significance.

### Ethical approval and informed consent

All experimental protocols and methods were approved by ethical guidelines (Ethical Committee for Animal Care and Use, Egypt), The Medical Research Ethics Committee of the National Organization for Drug Control and Research (NODCAR), Egypt under registration No 11/51 in 2022.

## Results

### Isolation and identification of bacterial isolate

**Figure 1:**
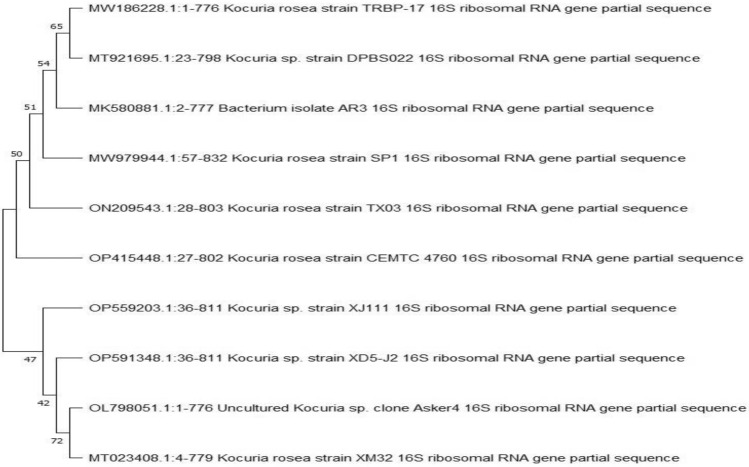
The phylogenetic tree of the partial sequence of 16S rRNA of the local isolate *Kocuria* sp. clone Asker4 respects closely related sequences available in Gen Bank databases.

A selection of thirty bacterial isolates was made from distinct marine materials, with the criteria for selection being their morphological variances observed on agar plates. Ultimately, a specific isolate (SM) was identified as a top producer of exopolysaccharide (EPS) based on its impressive yield of 5.3 g/L. The bacterial isolate exhibiting the highest level of potency was identified based on its morphological and physiological characteristics. These included being Gram-positive, possessing short rod-shaped structures, capable of generating spores, displaying motility, having a circular shape, exhibiting a smooth and mucous consistency, and displaying a yellow coloration. Furthermore, this isolate was found to be aerobic and tested positive for the catalase enzyme. Subsequently, validation was conducted utilizing a molecular approach, specifically targeting the 16s rRNA gene. The findings of this study indicate that isolate (SM) exhibits a close relationship with species belonging to the genus *Kocuria*. Consequently, the microorganism in question was identified as an uncharacterized strain of *Kocuria* sp., specifically designated as clone Asker4 and assigned the accession number OL798051.1. Figure 1 displays the phylogenetic tree representing the partial sequence of the 16S rRNA of the local isolate, Uncultured *Kocuria* sp. clone Asker4, in relation to closely related sequences identified in GenBank databases. The 16s rRNA gene of our isolate was used to identify and choose the *Kocuria* strains with the closest resemblance in their sequences. These selected strains were then connected to construct a phylogenetic tree that accurately represents their evolutionary relationships.

### Gene alignment for Kocuria sequence

**Figure 2:**
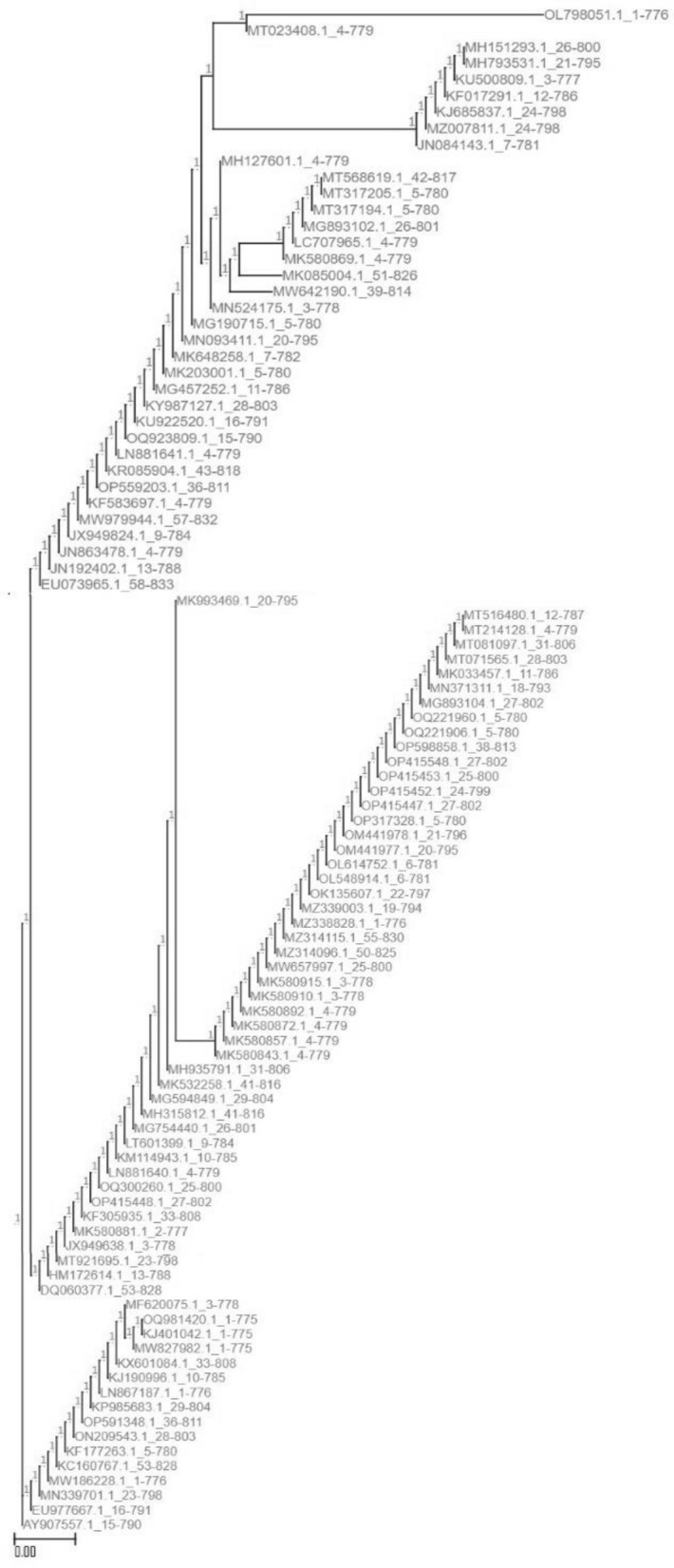
The phylogenetic trees of each of the genes.

**Figure 3:**
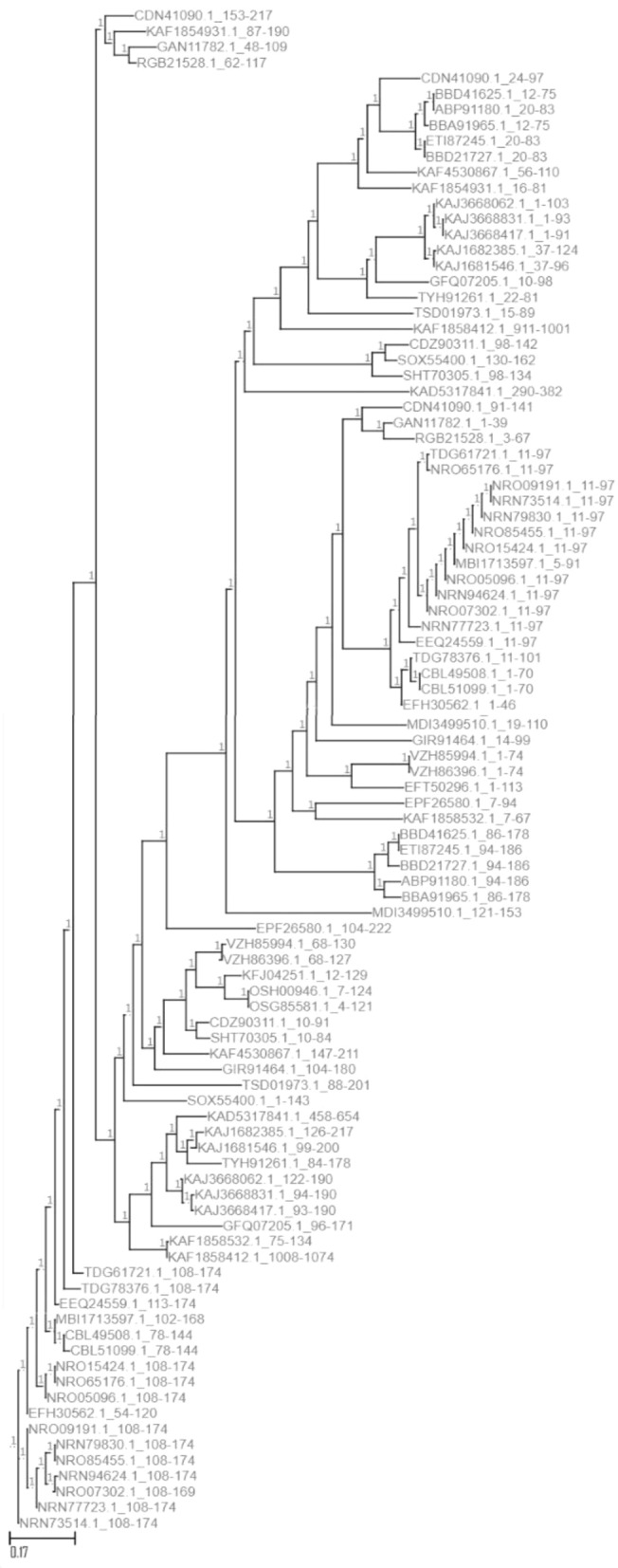
The top 50 protein alignments.

A blast alignment of encoded proteins took place using the balstx tool. The alignment gives the highest match to “hypothetical protein BN871_AB_00880 [*Paenibacillus* sp. P22]” (Accession: *CDN41090.1*) with the highest score and coverage and the lowest E-value. The phylogenetic trees (Fig. 2) of each of the genes and the top 50 protein alignments (Fig. 3) show the distance similarities and dissimilarities among the hits along with their hierarchical structures.

#### Modeling the 3D structure

The *Kocuria* gene translated sequence alignment hit a partial match with 3 different subsequences (153–217, 24–97, and 91–141, respectively) in the selected protein “hypothetical protein BN871_AB_00880 [*Paenibacillus* sp. P22]”. Each of the three subsequences undergoes 3D protein structure simulation and homology modeling separately.

### SWISS MODEL

#### Subsequence 1

**Figure 4:**
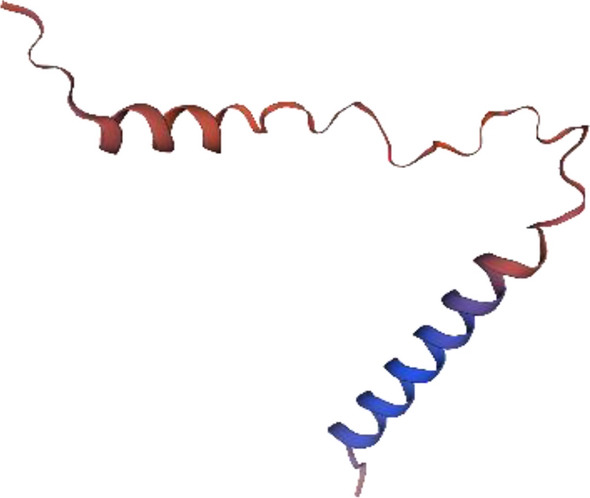
Subsequence 1 model 1 structure Ramachandran’s plot Mol Probity Score of 2.35 reflected the acceptable quality of the structure, which can be seen in the torsion angles that favored regions on the plot.

**Figure 5:**
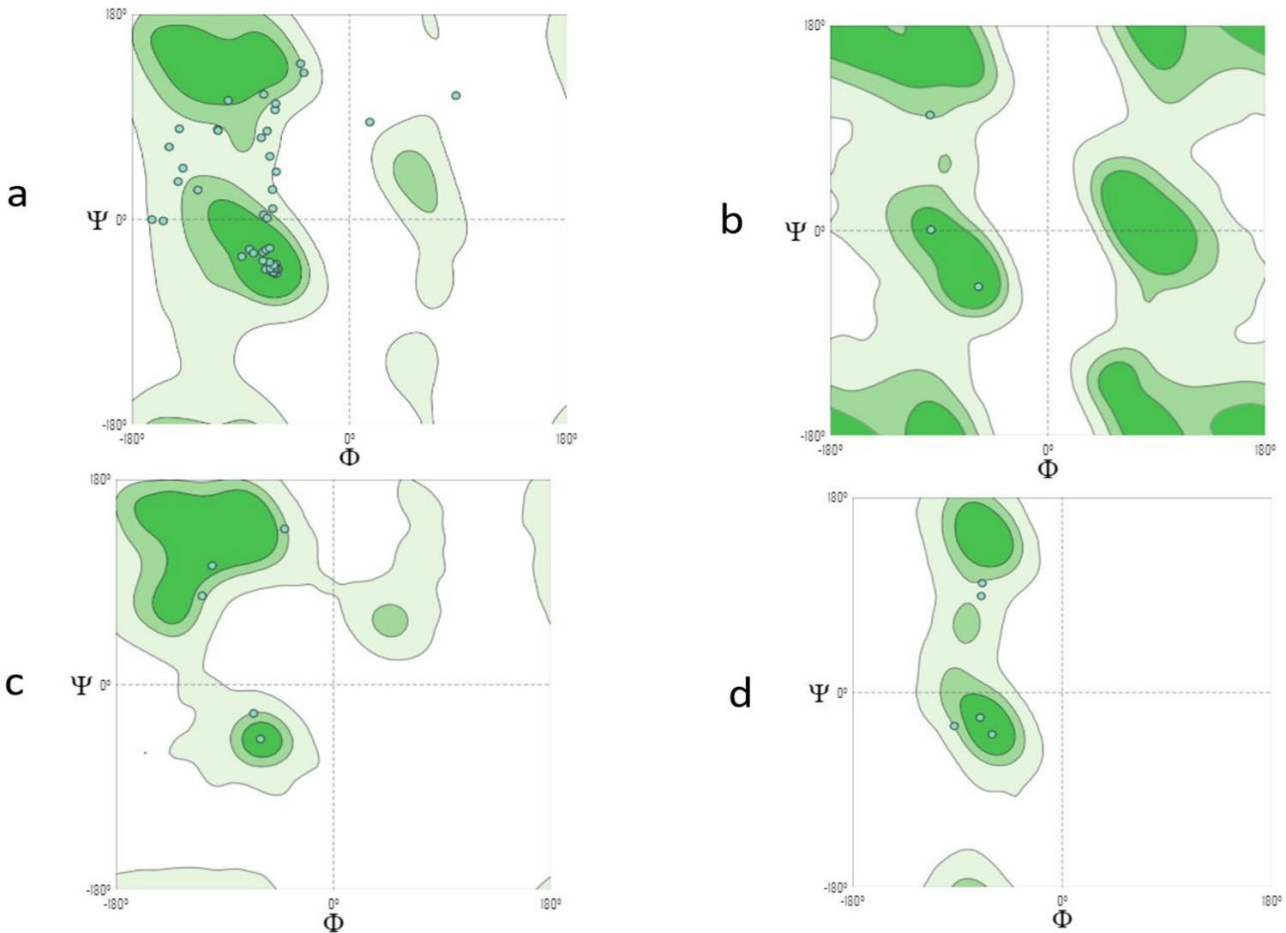
Ramachandran’s plot calculations on the 3D models of hypothetical protein BN871_AB_00880 [*Paenibacillus* sp. P22] subsequence 1 computed by the SWISS-MODEL web server to show the favored regions for backbone dihedral angles against amino acid residues in protein structure (**a**) General (no proline or glycine); (**b**) Glycine Only; (**c**) Pre-proline Only; (**d**) Proline Only.

Three models had been built, of which model 1 (Fig. 4) showed the highest coverage (97%), and sequence identity (74.6%) was selected and assessed with Ramachandran’s plot (Fig. 5). https://swissmodel.expasy.org/interactive/5gwExm/.

#### Subsequence 2

**Figure 6:**
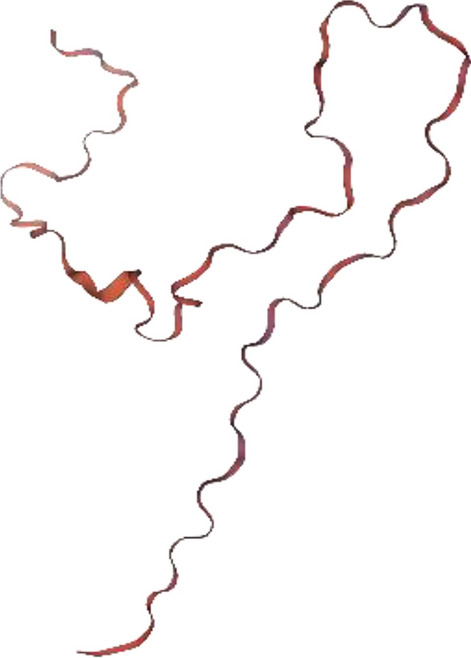
Subsequence 2 model 1 structure Ramachandran’s plot MolProbity Score.

**Figure 7:**
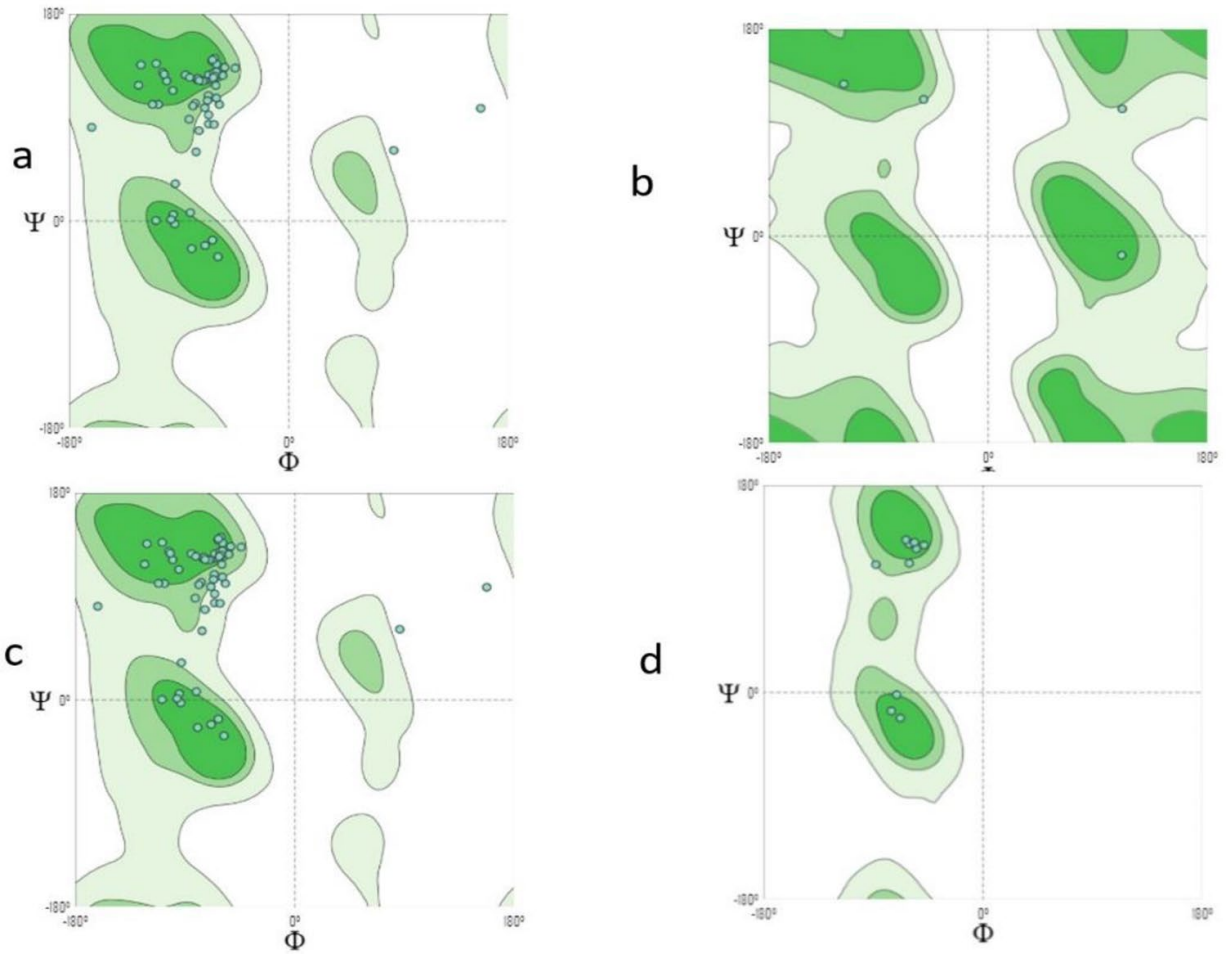
Ramachandran’s plot calculations on the 3D models of hypothetical protein BN871_AB_00880 [*Paenibacillus* sp. P22] subsequence 2 computed by the SWISS-MODEL web server to show the favored regions for backbone dihedral angles against amino acid residues in protein structure (**a**) General (no proline or glycine); (**b**) Glycine Only; (**c**) Pre-proline Only; (**d**) Proline Only.

Two models had been built, of which the first one (Fig. 6) showed a higher coverage (96%) and sequence identity (81.69%) was selected and assessed with Ramachandran’s Plot (Fig. 7). https://swissmodel.expasy.org/interactive/TtNYke/models/.

#### Subsequence 3

**Figure 8:**
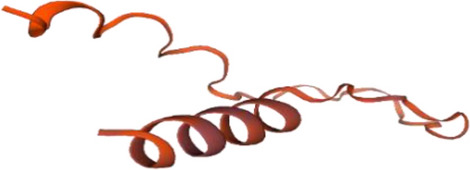
Subsequent 3 model structures.

**Figure 9:**
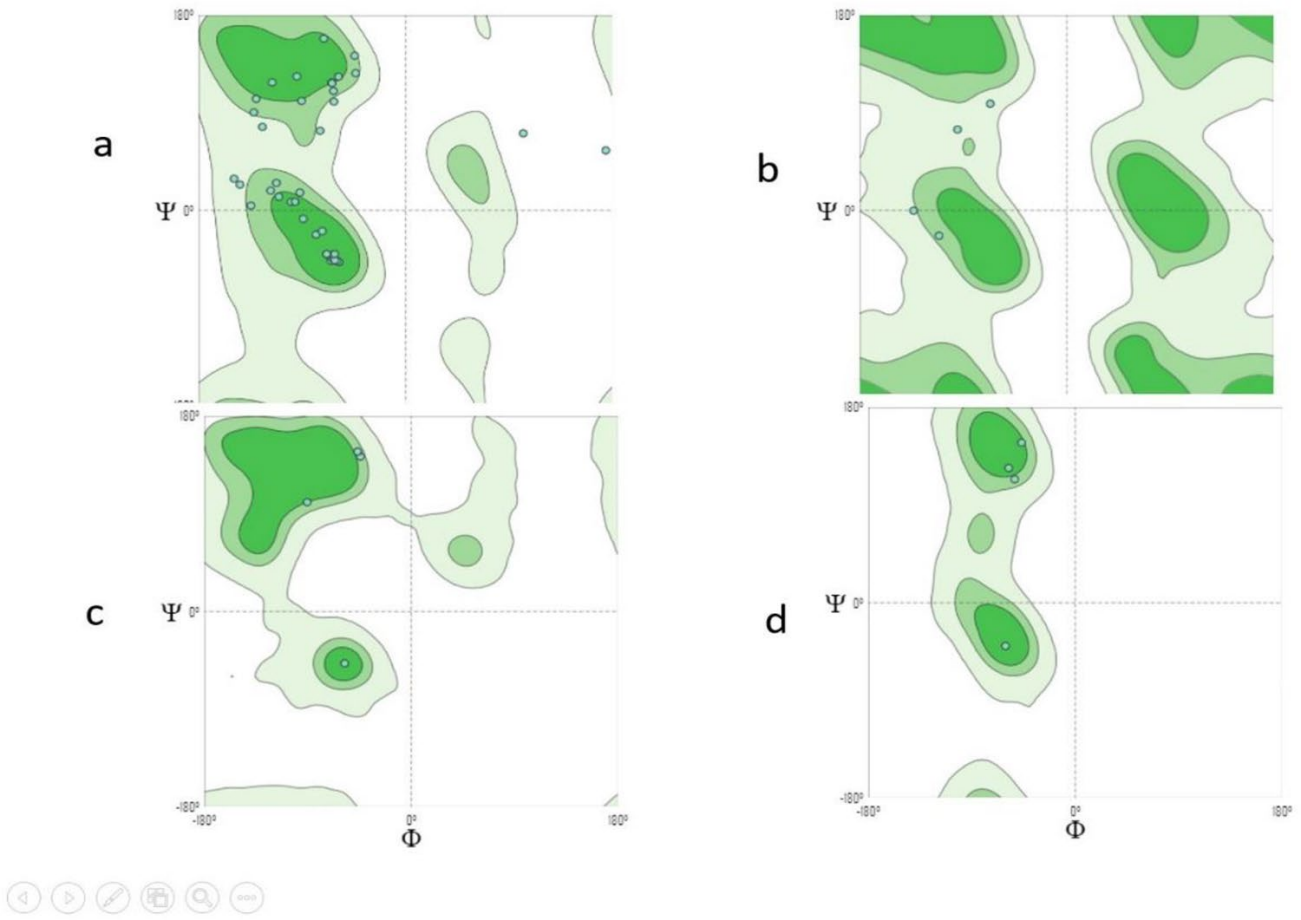
Ramachandran’s plot calculations on the 3D models of hypothetical protein BN871_AB_00880 [*Paenibacillus* sp. P22] subsequence 3 computed by the SWISS-MODEL web-server to show the favored regions for backbone dihedral angles against amino acid residues in protein structure (**a**) General (no proline or glycine); (**b**) Glycine Only; (**c**) Pre-proline Only; (**d**) Proline Only.

Only one model is generated (Fig. 8), which also expressed low quality with Ramachandran’s Plot (MolProbity Score 1.59) (Fig. 9). https://swissmodel.expasy.org/interactive/JWvYqE/models/.

#### I-TASSER

The I-TASSER models for the selected protein were ranked by C-score (confidence), and the top 5 are displayed. None of them showed significant confidence in the predicted structure (max C score =  − 4.16). Also, TM-score < 0.5 and RMSD > 2Å indicate poor similarity with the templates’ structure. The causes for that may be that the protein sequence is not well represented in the template library or is not flexible enough, or that the structure assembly simulations did not converge properly.

**Figure 10:**
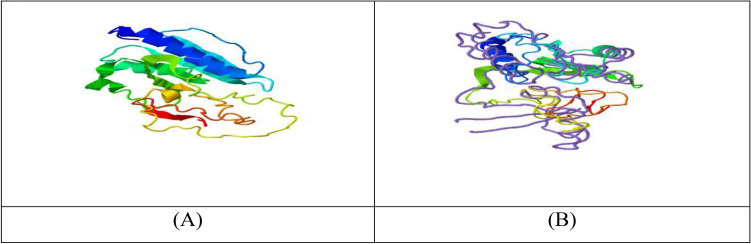
(**A**) i-tasser top c score model of *Kocuria species* top “hypothetical protein BN871_AB_00880 [*Paenibacillus* p22]”. (**B**) TM-align closest structural analogue (highest TM score).

The first one (Fig. 10A) then undergoes further structure similarity investigation with the protein PDB library by the *TM-align* structural alignment program, and the closest 10 identified structural analogs in PDB are obtained. *The cryo-EM structure of the A. thaliana Pol IV-RDR2 backtracked complex (pdb id: 7EUO)* (Fig. 10B) was the one with the highest TM-score.

#### COFACTOR

**Figure 11:**
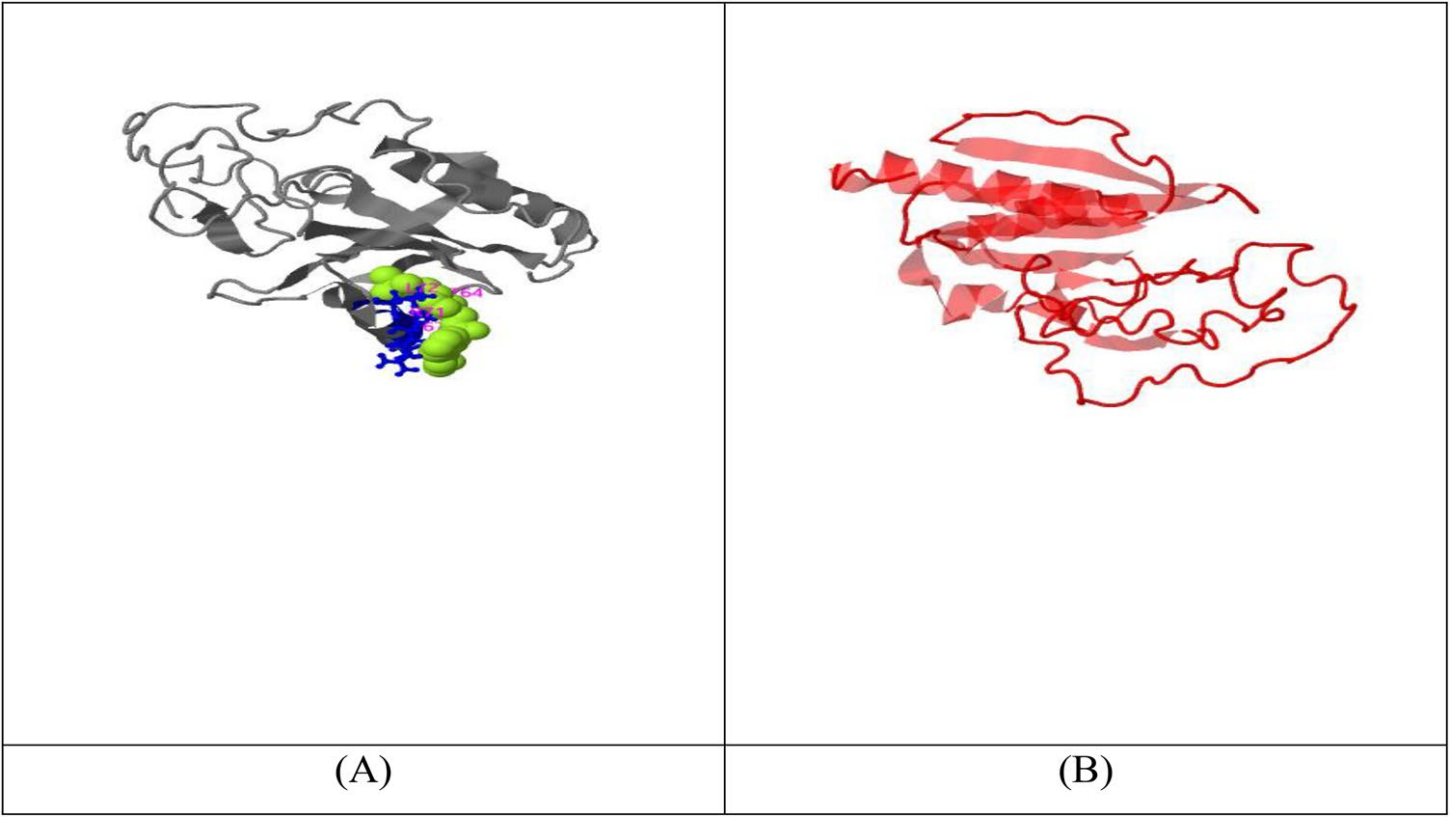
(**A**) X24 ligand with the highest C-score in COFACTOR; and (**B**) highest C and TM score for EC number prediction in COFACTOR.

COFACTOR is a tool of prediction for the structure of the top ligand binding site, enzyme commission (EC) number, and active sites*. X24 (pdb id: 2* × *24B)* is the ligand structure (Fig. 11) with the highest C-score (0.10), which is also not confident enough. The X24 binding site is shown in Fig. 11A. The elongation* complex of RNA polymerase II with a hepatitis delta virus-derived RNA stem loop (transferase) (pdb id: 2r93E) ***(**Fig. 11B) had the highest C and TM scores of EC number prediction. Moreover, gene ontology terms associated with each PDB structure are shown (link): http://zhanglab.ccmb.med.umich.edu/I-TASSER/output/S746151/.

#### COACH

The COACH software employs two comparison approaches, namely TM-SITE and S-SITE, to provide predictions of complementary ligand binding sites. These methods identify ligand-binding templates from the BioLiP protein function database. The aforementioned predictions will be integrated with outcomes obtained from several other methodologies, such as COFACTOR, FINDSITE, and ConCavity, in order to produce conclusive forecasts regarding ligand binding sites Table 2. These predictions can be accessed at the following link: https://seq2fun.dcmb.med.umich.edu/COACH/output/CH2099413790/.

**Table 2:** COACH complementary ligand binding site predictions using templates from the BioLiP protein function database.

Source	Top hit ligand	PDB hit	C-score	Structure
COACH	Nuc. Acid	2k1nD	0.07	
TM-SITE	CLA(1),GLY(1),III(1)	–	0.24	
S-SITE	NUC(2),UMP(2),GLA(1)	–	0.23	
COFACTOR	GLN	1jdbE	0.01	
FINDSITE	–	–	–	
ConCavity	–	–	0.08	

### Statistical modeling of EPSSM production

**Figure 12:**
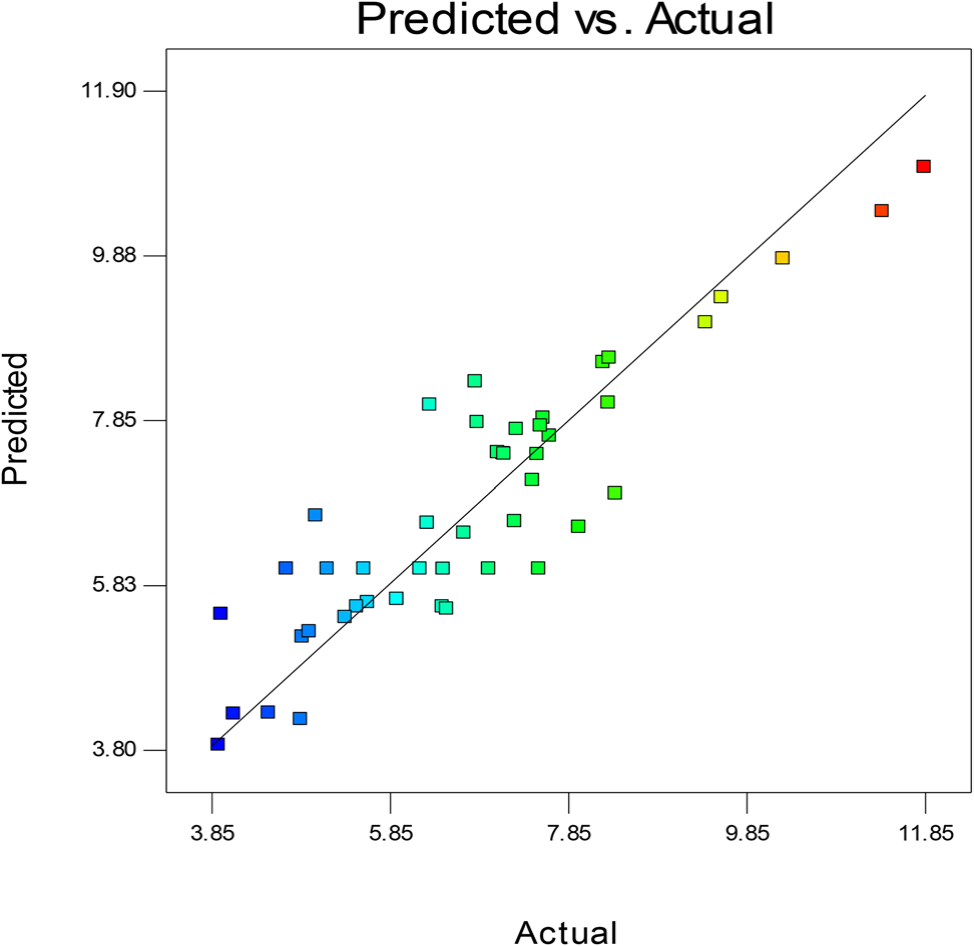
The relationship between experimental (actual) and predicted results.

Box–Behnken Design has been practically applied, and the results, along with predicted values and residuals, are shown in Table 3 and represented in Fig. 12. The results showed a good correlation coefficient between the actual and predicted results (R^2^ = 0.81).

**Table 3:** Actual and predicted results of polysaccharide production.

Run	Polysaccharide production (g/l)		
	Actual	Predicted	Residual
1	5.57	6.021667	− 0.45167
2	6.69	6.460417	0.229583
3	8.31	8.056667	0.253333
4	3.97	5.464583	− 1.49458
5	7.53	6.021667	1.508333
6	6.46	6.019167	0.440833
7	10.27	9.829167	0.440833
8	7.51	7.424167	0.085833
9	7.13	7.433333	− 0.30333
10	4.86	4.168333	0.691667
11	3.94	3.854167	0.085833
12	5.49	5.554167	− 0.06417
13	7.98	6.531667	1.448333
14	7.28	7.73	− 0.45
15	6.2	6.021667	0.178333
16	4.5	4.246667	0.253333
17	4.96	5.249167	− 0.28917
18	6.28	6.583333	− 0.30333
19	6.84	7.817083	− 0.97708
20	6.45	5.554167	0.895833
21	5.36	5.424167	− 0.06417
22	6.5	5.529167	0.970833
23	5.03	6.673333	− 1.64333
24	7.55	7.774583	− 0.22458
25	8.39	6.941667	1.448333
26	7.14	7.427917	− 0.28792
27	6.97	6.021667	0.948333
28	7.65	7.649167	0.000833
29	6.82	8.315	− 1.495
30	9.4	9.044167	0.355833
31	7.07	7.445	− 0.375
32	4.11	4.238333	− 0.12833
33	8.25	8.553333	− 0.30333
34	5.16	6.021667	− 0.86167
35	4.88	5.183333	− 0.30333
36	5.61	5.609167	0.000833
37	9.58	9.350417	0.229583
38	4.7	6.021667	− 1.32167
39	5.94	5.647083	0.292917
40	7.26	6.603333	0.656667
41	8.32	8.607917	− 0.28792
42	7.46	7.104167	0.355833
43	7.58	7.869167	− 0.28917
44	11.85	10.95417	0.895833
45	6.31	8.03	− 1.72
46	11.38	10.40917	0.970833

**Figure 13:**
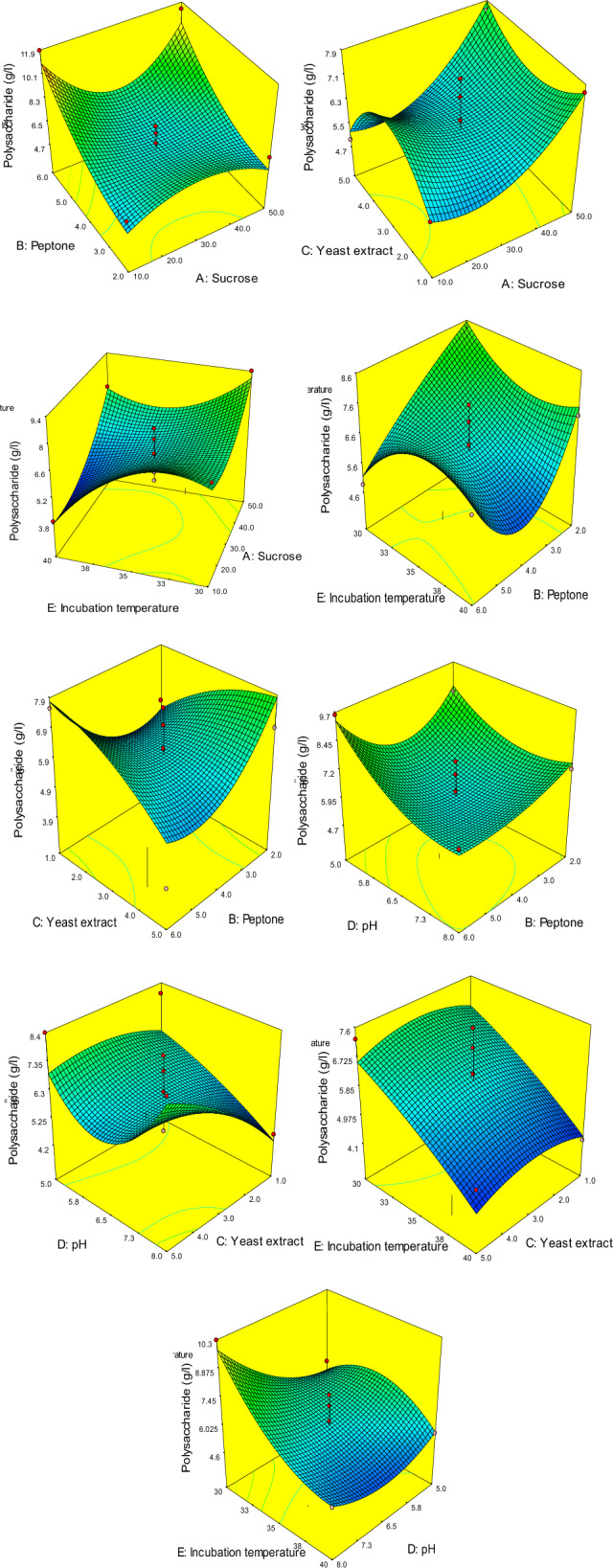
3D plotting of the variables and their effect on polysaccharide production, showing factor-factor interactions.

The data analysis of variance (ANOVA) resulted in a significant model (*P* value = 0.0064) and E, A^2^, B^2^, A^2^B as significant model terms (Table 4). Furthermore, the results showed that the model “lack-of-fit” was not significant, which means that the actual results match the predicted data of the model. Finally, the model “Adequate Precision” value was 7.679, which means that the model can be used to navigate the model space. The model resulted in the following model equation to numerically represent EPSSM production at predefined values of the variables (factors). The effect of the variables on EPSSM production along with factor-factor interactions is represented in 3D-plotting Fig. 13.

**Table 4:** Analysis of variance (ANOVA) of Box–Behnken results.

Source	Sum of squares	df	Mean square	F-value	*p* Value Prob > F
Model	118.4751	26	4.556735	3.128665	0.0064
A-sucrose	0.16245	1	0.16245	0.111539	0.7421
B-peptone	0.025312	1	0.025312	0.01738	0.8965
C-yeast extract	0.0147	1	0.0147	0.010093	0.9210
D-pH	0.68445	1	0.68445	0.469945	0.5013
E-incubation temperature	17.78768	1	17.78768	12.21306	0.0024
AB	0.0676	1	0.0676	0.046414	0.8317
AC	0.0841	1	0.0841	0.057743	0.8127
AE	0.664225	1	0.664225	0.456058	0.5076
BC	5.0176	1	5.0176	3.445096	0.0790
BD	0.731025	1	0.731025	0.501923	0.4873
BE	1.5876	1	1.5876	1.09005	0.3096
CD	2.89	1	2.89	1.984281	0.1751
DE	3.8809	1	3.8809	2.664635	0.1191
A^2^	8.800152	1	8.800152	6.042204	0.0237
B^2^	10.28975	1	10.28975	7.064967	0.0155
C^2^	1.626206	1	1.626206	1.116557	0.3039
D^2^	6.367424	1	6.367424	4.371888	0.0502
E^2^	0.249752	1	0.249752	0.17148	0.6834
A^2^B	18.3925	1	18.3925	12.62834	0.0021
AC^2^	4.558817	1	4.558817	3.130094	0.0929
AE^2^	6.161067	1	6.161067	4.230203	0.0537
B^2^D	1.401667	1	1.401667	0.962388	0.3389
B^2^E	4.972969	1	4.972969	3.414452	0.0803
BE^2^	2.660004	1	2.660004	1.826365	0.1924
CD^2^	3.5643	1	3.5643	2.447257	0.1342
DE^2^	3.920417	1	3.920417	2.691767	0.1173
Residual	27.6725	19	1.456447		
Lack of fit	21.77301	14	1.555215	1.318094	0.4060
Pure error	5.899483	5	1.179897		
Cor total	146.1476	45			

Polysaccharide (g/l) =  − 330.5238773 + 4.36071875 * A − 12.31421875 * B + 12.97534722 * C + 46.30814815 * D + 17.24225 * E − 0.20021875 * A * B − 0.0944375 * A * C − 0.208725 * A * E − 0.28 * B * C + 0.824166667 * B * D + 0.94625 * B * E − 2.865555556 * C * D − 2.394666667 * D * E − 0.010620833 * A^2^ − 1.19625 * B^2^ − 0.598229167 * C^2^ − 0.347037037 * D^2^ − 0.228233333 * E^2^ + 0.003282813 * A^2^ * B + 0.01634375 * A * C^2^ + 0.00304 * A * E^2^ − 0.120833333 * B^2^ * D + 0.064375 * B^2^ * E − 0.019975 * B * E^2^ + 0.242222222 * C * D^2^ + 0.032333333 * D * E^2^.

*Where*: A: Sucrose; B: Peptone; C: Yeast extract; D: pH; E: Incubation temperature.

### Numerical optimization of EPSSM production

EPSSM production has been numerically maximized at the studied levels of the variables and resulted in maximum EPSSM production (12.95 g/l) at the following conditions: 31.8 g/l, sucrose; 2.4 g/l, peptone; 4.9 g/l, yeast extract; 7.9 pH; and 30 °C, incubation temperature with maximum desirability.

### EPSSM isolation and purification

The EPSSM production reached a value of 5.3 g/l in the medium after a 5-day period of development. The primary fraction was obtained through the use of ethanol precipitation following fractionation of the crude EPSSM. This was achieved by employing a two-volume ethanol method. The EPSSM has been collected for subsequent evaluation of its structural characteristics. The substance materialized in the form of a white, powdery substance, eliciting an unfavorable reaction when subjected to the Bradford test. The absence of absorption at 260 and 280 nm in the UV spectra indicated the absence of RNA, DNA, and/or protein.

### Purification and fractionation of the main fraction by DEAE-cellulose column

Most of the partially purified EPSSM that came from a two-volume ethanol treatment were put through chromatography on a DEAE-cellulose column. The carbohydrate concentrations of each eluted aliquot (1 mL/1 min) were measured. The application of any subsequent elution was contingent upon the absence of carbs in the final aliquot of the prior elution. The elution of the fraction not bound to the tertiary amine of DEAE-cellulose can be achieved using distilled water, whereas the elution of acidic or sulfated polysaccharide that are attached to the DEAE-cellulose can be achieved using salt solutions of appropriate polarity, based on the acidity level of the sulfated and uronic groups present on the polysaccharide molecules. It was noticed that the main fraction was effectively eluted into four subfractions. The activity of these subfractions was detected as antioxidant. The promising fraction (EPSSM) was pooled and dialyzed against deionized water, then lyophilized for further studies.

### Chemical structure of EPSSM

The EPSSM sample was found to have a uronic acid content of 13.9%, determined via colorimetric m-hydroxydiphenyl analysis. Additionally, the sample had a sulphate content of 13.63%. HPLC determined the molar ratio of monosaccharides in the EPSSM hydrolysate, in which fructose: glucouronic acid: xylose with 2.0: 0.5: 1.0, respectively. The calculation of the molecular weight of EPSSM was performed. The EPSSM Mw was determined to be 1.18 × 10^5^ g/mol, while Mn was determined to be 1.05 × 10^5^ g/mol. The polydispersity index (*Mw/Mn*) is a quantitative measure used to assess the breadth of the molecular weight distribution, with a value of 1.12.

**Figure 14:**
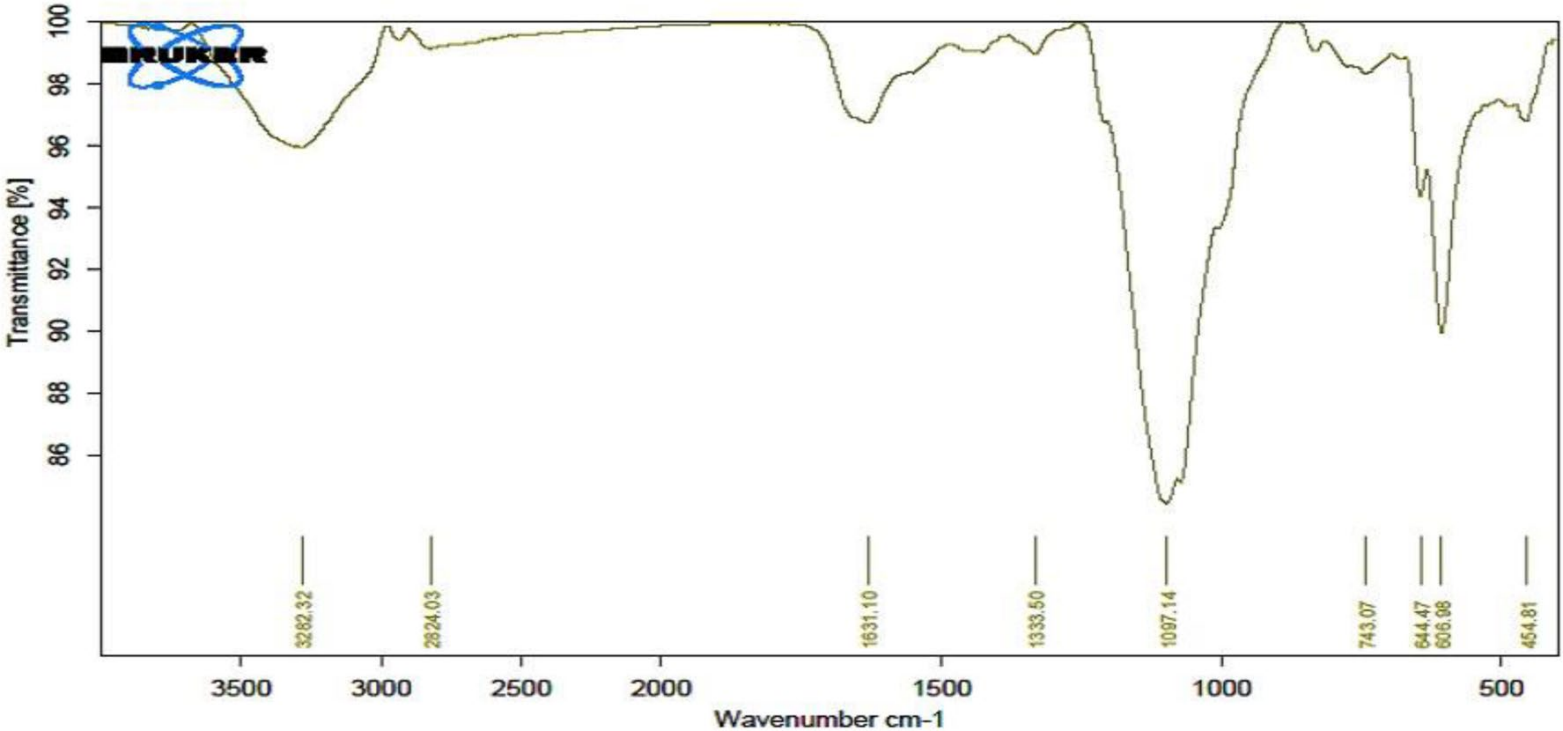
FT-IR spectrum of the EPSSM.

According to the FT-IR assessment presented in Fig. 14, it is suggested that the EPSSM may potentially be associated with the α-anomeric configuration and that had sulfate groups, hydroxyl group, secondary and primary (CH_2_), glycosidic bond (C–O–C), and the α-pyranose.

### EPSSM antioxidant activity

**Figure 15:**
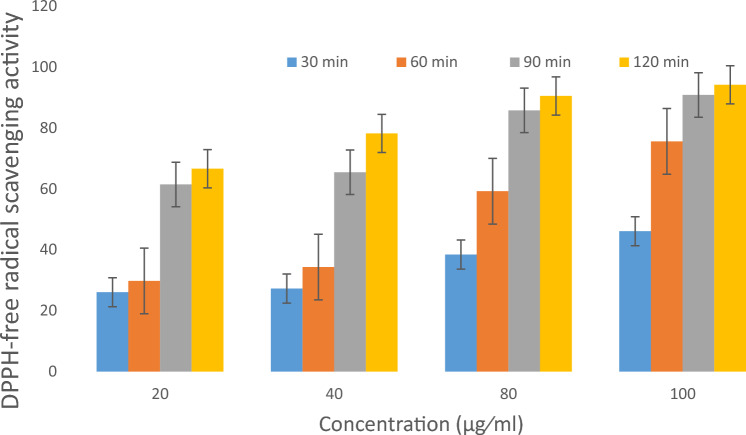
The DPPH radical-scavenging activities of EPSSM.

In order to assess the effectiveness and efficiency of EPSSM in enhancing in-vitro antioxidant activities, the DPPH scavenging rate was seen to exhibit a proportionate increase with the increasing concentration of EPSSM ranging from 20 to 100 µg/mL across various time intervals (30, 60, 90, 120 min). The EPSSM exhibited the most significant DPPH-free radical scavenging activity, with an estimated value of 94.13% at a concentration of 100 µg/mL after a duration of 120 min. Figure 15 demonstrates that the DPPH-free radical scavenging activity was found to be the lowest at a concentration of 20 µg/mL after 120 min, with an estimated value of 66.59%. The use of power activity reduction in EPSSM results in a value of 1.439 ± 0.015266 at a concentration of 500 µg/mL, as reported in Table 5.

**Table 5:** Antioxidant activity by reducing power.

Concentration (µg/ml)	Antioxidant
100	0.874 ± 0.007022
200	0.899 ± 0.008937
300	0.971 ± 0.011128
400	1.014 ± 0.076368
500	1.439 ± 0.015266

### EPSSM-repressed induced inflammatory responses and oxidative stress induced by carrageenan injection in rats

**Figure 16:**
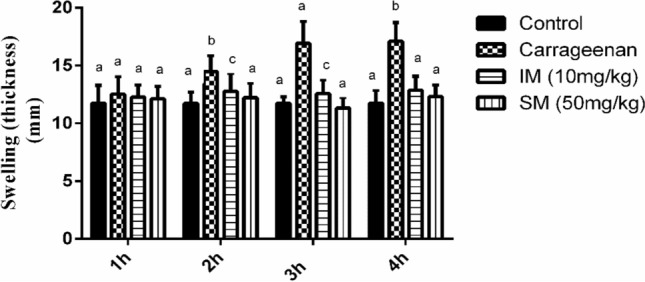
Anti-inflammatory effects of EPSSM on carrageenan-induced hind paw edema in rats. Values are expressed as means ± SEM of six rats per group. Values in the same column not sharing the same superscript letters were significantly different (*P* < 0.05), IM: indomethacin.

The current work aimed to examine the potential anti-inflammatory effects of EPSSM through the utilization of the carrageenan-induced hind paw edema model. The findings of our study provide compelling evidence of the anti-inflammatory and antioxidant efficacy of EPSSM. In the carrageenan-induced paw edema model in comparison with indomethacin as a reference drug. It was observed that carrageenan injection directly increased right paw thickness measured at 1, 2, 3, and 4 h, as shown in Fig. 16. In meanwhile, administration of Indomethacin at a dosage of 10 mg/kg across the entire duration of the experiment resulted in a significant suppression of induced paw swelling (*P* < 0.05), with the highest level of inhibition observed 2 h following injection. As illustrated in Fig. 16, it was observed that EPSSM (50 mg/kg) diminished.

Effects of EPS on anti-inflammatory responses paw edema at 2, 3, and 4 intervals.

**Figure 17:**
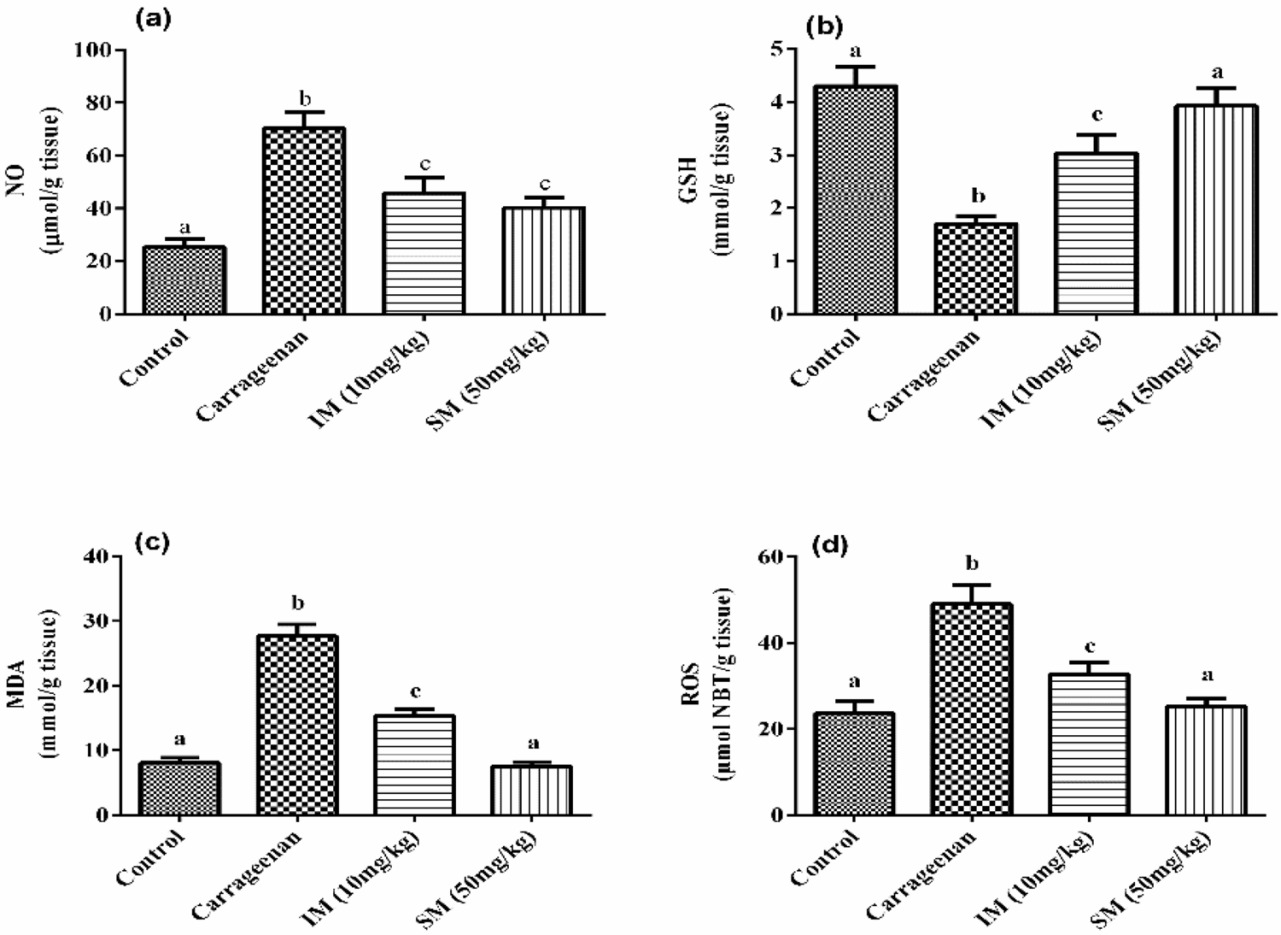
Effect of EPSSM on changes in MDA, GSH, NO and ROS levels in carrageenan-induced paw edema in rats. Values are expressed as means ± SE of six rats per group. Values in the same column not sharing the same superscript letters were significantly different (*P* < 0.05), IM: indomethacin.

**Figure 18:**
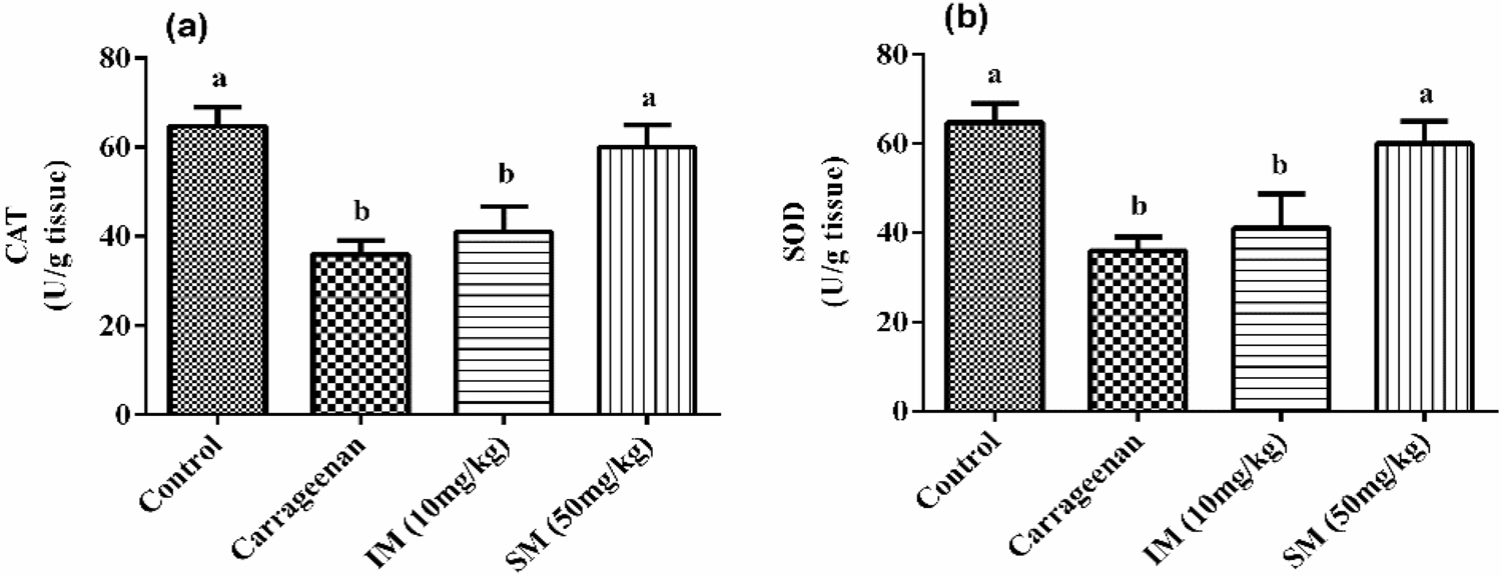
Effect of EPSSM on changes in CAT and SOD activities in carrageenan-induced paw edema in rats. Values are expressed as means ± SE of six rats per group. Values in the same column not sharing the same superscript letters were significantly different (*P* < 0.05), IM: indomethacin.

**Figure 19:**
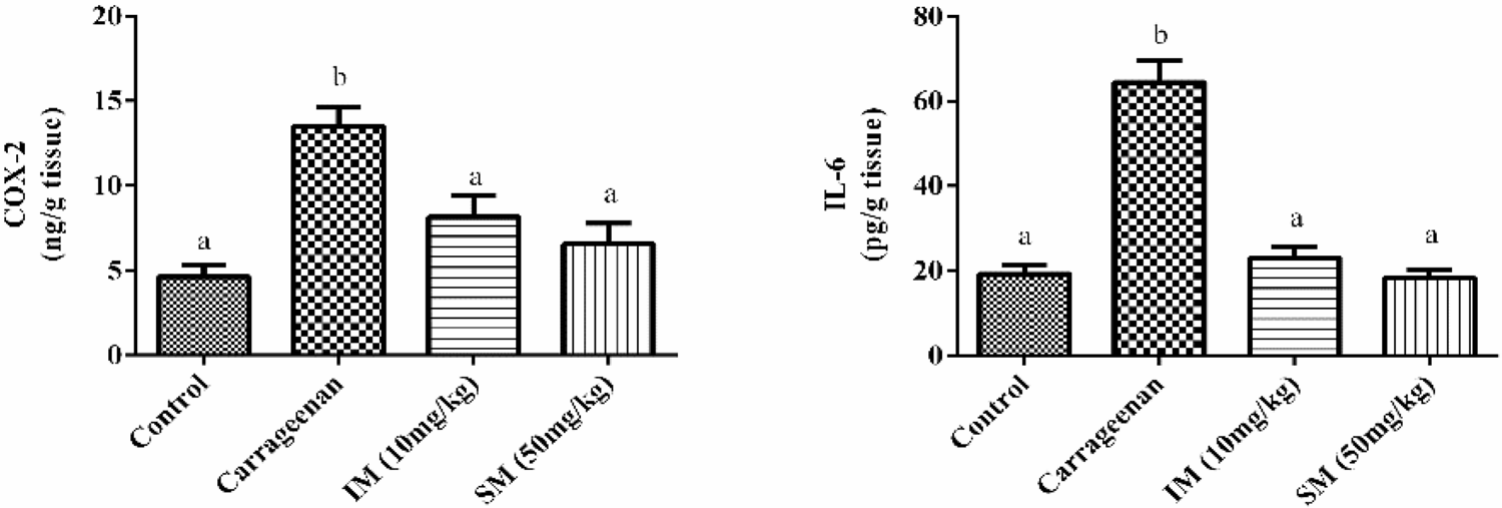
Effects of EPSSM on IL-6 and COX-2 protein expressions of edema paw in rats Values are expressed as means ± SE of six rats per group. Values in the same column not sharing the same superscript letters were significantly different (*P* < 0.05), IM: indomethacin.

The effect of EPSSM on carrageenan-induced paw edema was illustrated by the increased time-dependent paw thickness in a cascading manner after 1 h and was observed to be maximum following 4 h. However, after EPSSM injection in comparison with indomethacin, paw edema (*P* < 0.005) was gradually observed to decrease at 2, 3, and 4 h, respectively. Thereby, the results obtained from the experiment indicate that EPSSM plays a crucial function in protecting against the excessive inflammation triggered by the infusion of carrageenan. Since inflammatory-mediated processes are thought to be a chain of physiological responses, like trauma and infection, to inflammatory stimuli in humans, it is thought that carrageenan-induced edema in rats is a two-stage process. The first (1h) stage includes instant histamine and serotonin release. Meanwhile, the second stage (≥ 1 h) can be related to the release of prostaglandins. The antioxidant SOD, CAT, MDA, GSH, ROS, NO, COX-2, and IL-6 activities were all detected in paw tissues among all the groups to find out the antioxidant and anti-inflammatory activities of EPSSM, as shown in Figs. 17, 18 and 19. Our results revealed that EPSSM significantly increased the GSH, SOD, and catalase activities in the carrageenan-induced paw edema when compared with the indomethacin-treated group and the control group, as shown in Figs. 17 and 18. The carrageenan hindered GSH, CAT, SOD, and antioxidant activities in induced rats. Meanwhile, the treatment of rats with EPS reversed these changes due to the cytoprotective and antioxidant activities of EPSSM, which can be related to the increased antioxidant enzyme activities to scavenge elevated free radicals and lipid peroxides. An observed decrease in ROS and MDA levels was detected following the treatment with EPSSM and indomethathin in the carrageenan-induced rats, with a more relevant decrease in the EPSSM-treated groups when compared with the control group. We find that a relevant decrease in ROS and MDA associated with a relevant increase in GSH, SOD, and Cat following EPS treatment, which can be related to the nature of the exopolysaccharide structure and their antioxidant properties, as shown in Figs. 17 and 18. The inflammatory-mediated cascades as a drawback of carrageenan injection are initially mediated by Toll-like receptor-4 (TLR-4). Once TLR-4 is mediated, exaggerated cytokines such as nitric oxide (NO) and pro-inflammatory cytokines such as COX-2 and IL-6 are released, as detected in our observed results as shown in Fig. 19.

## Discussion

In the current study, we characterized a unique exopolysaccharide-type EPSSM isolated from *Kocuria* sp. clone Asker 4 with accession number OL798051.1. The study of the 16S rRNA gene sequence is a robust and precise approach for elucidating evolutionary connections^35^. Several marine bacteria, including *Edwardsiella tarda, Alteromonas*, and *Paenibacillus polymyxa*, have the ability to produce EPSs^36^. In our findings, marine *Kocuria* sp. produced 5.3 g/l from the EPSSM and was then purified, fractionated, and analyzed as a glycosidic compound with unique chemical compositions that have a uronic acid and sulfate content. The EPSSM Mw was determined quantitatively similar to another study by Smiderle et al.^37^, Silveira et al.^38^ showed a significant antinociceptive effect of mushroom heteropolysaccharide in mice as an anti-inflammatory action. The functional groups included in the EPS chains play a crucial role in governing various biological processes^39^. The study found that EPSs are routinely observed in the EPSs of halophilic bacteria as well as in specific marine bacterial EPSs and the cellular walls of brown and red algae^40–42^. Sulfated EPSs possess considerable potential in the field of medicine due to their diverse range of bioactive characteristics^43,44^. The bands observed at a wavenumber of 3282.32 cm^−1^ exhibited band intensity indicative of OH axial deformation, which corresponded to the presence of both intermolecular and intramolecular hydrogen bonding^45^. The soft band seen at 2824.03 cm^−1^ in both the secondary and primary (CH_2_) bands was attributed to the axial deformation of the CH moiety^46,47^. The absorptions observed at around 1333.82 cm^−1^ were indicative of the bonding between CH_2_ and OH. The vibration-stretching of the glycosidic bond (C–O–C) resulted in a prominent absorption peak at 1097.14 cm^−1^^48^. Furthermore, the conformation of the glucosyl residue in α-pyranose form was determined based on the presence of a band at 743.07 cm^−1^. According to the FT-IR assessment presented in our study, it is suggested that the EPSSM may potentially be associated with the α-anomeric configuration^49^. We explored and studied the antioxidant activity of the EPSSM and the results exhibited strong DPHH-free radical scavenging activity. It was previously reported that the EPS free-radical scavenging activity can be considered as high and effective as ascorbic acid in a gradual dose-dependent manner^50–52^. This significantly observed activity can be related to the presence of various antioxidant compounds, including peptides, proteins, and microelements^11,51,53^. Additionally, EPSs were reported to consist of multiple monosaccharide compounds, including glucose, mannose, galactose, and glucuronic acid, which are considered reductive agents with a potent hidden aldehyde moiety^54^. These adjuvant components are suggested to exhibit potent synergistic antioxidant activities. Meanwhile, the EPSSM antioxidant properties scavenge free radicals and reduce the risk of ROS accumulation by potentially degrading superoxide anion and hydrogen peroxide^54,55^. We studied the anti-inflammatory biological activity of the EPSSM and observed results that exhibited edema-relieving potential and anti-inflammatory properties when compared with the indomethacin reference drug. This suggests that EPSSM could be considered an attractive candidate of choice as a safe natural product-derived agent for regulating oxidative stress and inflammation. It still remains unclear how EPSSM itself induces the expression of TLR4 and the release of proinflammatory cytokines. It can be suggested to conduct further studies to determine the exact mode of action of EPSSM. In another study, the anti-inflammatory activities of exopolysaccharides isolated from red marine algae were previously reported to have potential anti-inflammatory effects against carrageenan-induced rat paw edema^56^. The aforementioned inflammatory processes can be regarded as a transient occurrence, distinguished by a significant influx of neutrophils and the presence of several mediators, including histamines, bradykinins, serotonin, prostaglandins, and nitric oxide^57–59^. Anti-inflammatory activities of the body are considered complicated biological reactions to diseases, wounds, irritations, and harm to cells. Inflammation acts as a cascading predisposing factor for multiple illnesses, including rheumatoid arthritis, asthma, neurodegenerative diseases, inflammatory intestinal disease, and cancer^60,61^. Several pro-inflammatory-mediated factors, such as IL-6 and COX-2, were previously reported to be induced during the propagation of the inflammatory process^60,61^. The present study aimed to assess the anti-inflammatory effects of EPSSM in a rat model of carrageen-induced paw edema^62^. The presence of edema in the paw is a consequence of the inflammatory mediators that include TLR-4, TNF-alpha, COX-2, and IL-6^63,64^. Since inflammatory-mediated processes are thought to be a chain of physiological responses, like trauma and infection, to inflammatory stimuli in humans, it is thought that carrageenan-induced edema in rats is a two-stage process. The first (1h) stage includes instant histamine and serotonin release. Meanwhile, the second stage (≥ 1 h) can be related to the release of prostaglandins^63–65^. In accordance with our results, previous studies have reported that isolated EPSSM has potent anti-inflammatory actions due to its inhibitory actions on inflammatory and oxidative mediator factors such as COX-2, Il-6, MDA, ROS, and NO^66,67^. In agreement with our observed results, carrageenan hindered GSH, CAT, SOD, and antioxidant activities in induced rats. Meanwhile, the treatment of rats with EPS reversed these changes due to the cytoprotective and antioxidant activities of EPSSM, which can be related to the increased antioxidant enzyme activities to scavenge elevated free radicals and lipid peroxides. An observed decrease in ROS and MDA levels was detected following the treatment with EPSSM and indomethathin in the carrageenan-induced rats, with a more relevant decrease in the EPSSM-treated groups when compared with the control group. The previously elevated levels of SOD, Cat, and GSH in the EPSSM-treated group are suggested to be the main factor in alleviating the MDA and ROS elevated levels. In agreement with our findings Handy and Moore^65^, Sun et al.^54^, Du et al.^67^ reported a relevant decrease in ROS and MDA associated with a relevant increase in GSH, SOD, and Cat following EPS treatment, which can be related to the nature of the exopolysaccharide structure and their antioxidant properties**.** These exaggerated cytokines are pro-inflammatory cytokines that directly activate NF-κB, resulting in progressive inflammatory conditions such as tissue damage, asthma, and rheumatoid arthritis^63,67^. Sun et al.^54^, Murofushi et al.^63^ reported in agreement with our results, EPS has potential antioxidant, DPPH free radical scavenging, and anti-inflammatory activities by stimulating immunomodulatory action, enhancing phagocytosis and macrophage activity, and promoting the secretion of COX-2, IL-6, and IL-10 associated with a relevant decrease in NO, MDA, and ROS. Among both treatment groups, indomethacin was the reference standard group, and EPSSM showed a relevant decrease in induced inflammatory edema, inflammatory-mediated factors including COX-2 and TNF-alpha, and oxidative stress including MDA, ROS, and NO. A significant increase in anti-oxidative factors such as Cat, SOD, and GSH was also observed. However, among these observed improvements among both treatment groups, a more significant improvement was observed among the EPSSM-treated group when compared with the indomethacin reference group and control group. In accordance with our observed results. The efficiency of isolated EPS as a more potent antioxidant and anti-inflammatory agent when compared with the indomethacin reference drug. This indicates the efficiency and efficacy of administering EPSSM in alleviating severe inflammation and exaggerated oxidative stress^64,68^.

## Data Availability

All data generated or analyzed during this study are included in this published article.
